# The impact of wall thickness and curvature on wall stress in patient-specific electromechanical models of the left atrium

**DOI:** 10.1007/s10237-019-01268-5

**Published:** 2019-12-04

**Authors:** Christoph M. Augustin, Thomas E. Fastl, Aurel Neic, Chiara Bellini, John Whitaker, Ronak Rajani, Mark D. O’Neill, Martin J. Bishop, Gernot Plank, Steven A. Niederer

**Affiliations:** 1 Department of Biomedical Engineering, King’s College London, London, UK; 2 Department of Mechanical Engineering, University of California, Berkeley, Berkeley, USA; 3 Department of Bioengineering, Northeastern University, Boston, USA; 4 Department of Cardiology, Guy’s and St Thomas’ Hospitals, London, UK; 5 Gottfried Schatz Research Center: Division of Biophysics, Medical University of Graz, Graz, Austria; 6 BioTechMed-Graz, Graz, Austria

**Keywords:** Left atrium, Cardiac mechanics, Finite element simulation, Patient-specific modeling, Wall stress

## Abstract

The left atrium (LA) has a complex anatomy with heterogeneous wall thickness and curvature. The anatomy plays an important role in determining local wall stress; however, the relative contribution of wall thickness and curvature in determining wall stress in the LA is unknown. We have developed electromechanical finite element (FE) models of the LA using patient-specific anatomical FE meshes with rule-based myofiber directions. The models of the LA were passively inflated to 10mmHg followed by simulation of the contraction phase of the atrial cardiac cycle. The FE models predicted maximum LA volumes of 156.5 mL, 99.3 mL and 83.4 mL and ejection fractions of 36.9%, 32.0% and 25.2%. The median wall thickness in the 3 cases was calculated as $1.32\, \pm \,0.78$ mm, $1.21\, \pm \,0.85$ mm, and $0.74\,\pm \,0.34$ mm. The median curvature was determined as $0.159\,\pm \,0.080$ $\hbox {mm}^{-1}$, $0.165\,\pm \,0.079\,\hbox {mm}^{-1}$, and $0.166\,\pm \,0.077\,\hbox {mm}^{-1}$. Following passive inflation, the correlation of wall stress with the inverse of wall thickness and curvature was 0.55–0.62 and 0.20–0.25, respectively. At peak contraction, the correlation of wall stress with the inverse of wall thickness and curvature was 0.38–0.44 and 0.16–0.34, respectively. In the LA, the 1st principal Cauchy stress is more dependent on wall thickness than curvature during passive inflation and both correlations decrease during active contraction. This emphasizes the importance of including the heterogeneous wall thickness in electromechanical FE simulations of the LA. Overall, simulation results and sensitivity analyses show that in complex atrial anatomy it is unlikely that a simple anatomical-based law can be used to estimate local wall stress, demonstrating the importance of FE analyses.

## Introduction

Atrial fibrillation (AF) is a prevalent and progressive disease, characterized by chaotic electrical activation of the atria (Kirchhof et al. 2016). Early detection and treatment of AF are associated with improved patient outcome and reduced stroke risk (Keach et al. 2015). While AF is an electrophysiological pathology, the risk of developing AF is markedly increased with hypertension, mitral regurgitation, mitral stenosis and aortic stenosis, which increase the mechanical loading on the atria (Benjamin et al. 1994; Iung et al. 2018; Widgren et al. 2012). The changes in mechanical loading of cardiac tissue can activate fibroblasts, leading to an increased fibrotic burden, which might contribute to the initiation and sustenance of AF (Marrouche et al. 2014; Dzeshka et al. 2015).

In the heart, mechanical quantities, such as stress and strain, have previously been used to drive models of growth and remodeling (Kerckhoffs et al. 2012; Rodriguez et al. 1994). While strain can be measured directly from clinical images (Blume et al. 2011), stress must be calculated using a mathematical model (Yin 1981), accounting for the anatomy, micro-structure and material properties of the atria. In the left ventricle, the wall stress can be approximated using the Law of Laplace (Valentinuzzi and Kohen 2011), in which wall stress is proportional to the radius of curvature (inverse of curvature) and inversely proportional to the wall thickness. The Law of Laplace assumes that the wall of the heart is thin relative to the radius of curvature. Due to the thin wall of the atria, the Law of Laplace may provide a reasonable approximation of atrial stress. In the atria, both the wall thickness (Bishop et al. 2016) and curvature (Ahmed et al. 2006) vary across the surface. However, their relative influence on wall stress remains unknown. In addition, other attributes of the atria including the complex anatomy, fiber structures, boundary conditions and active contraction play a role in determining the wall stress and are not accounted for the in the Law of Laplace, potentially limiting its applicability in the atria.

Previous models of human atrial mechanics have assumed a homogeneous wall thickness (Moyer et al. 2015; Hunter et al. 2012). Models of atrial mechanics that accounted for regional variations in thickness were derived from cadaveric data sets and might not reflect the *in-vivo* anatomy of the atria (Adeniran et al. 2015). Recent developments in computed tomography (CT) image analysis now allow the generation of anatomically detailed geometric models of the LA that account for varying wall thickness derived from clinical scans (Bishop et al. 2016). To determine if local wall stress analysis in the atria requires patient-specific wall thickness, we investigated if wall thickness is an important factor in determining local atrial stress or if the curvature, i.e., the endocardial surface shape, was the dominant factor.

In this study, we first describe an electromechanical modeling framework for simulating active contraction in the LA. Secondly, we perform representative finite element (FE) simulations of the passive inflation and active contraction in the LA. Thirdly, we calculate the wall thickness and curvature across the endocardial LA surface. Finally, we compare the correlation between the 1st principal stress with wall thickness and curvature to identify the more prominent metric.

## Methods

### Personalized model generation

We focused our modeling efforts on the LA, which plays a more dominant role in AF compared to the right atrium (Kirchhof et al. 2016). The FE simulations were performed on 3 publicly available LA anatomical models developed from CT angiography images, that include a description of the endocardial and epicardial myofiber distributions (Fastl et al. 2018). Atrial fibers were represented by two distinct layers, consistent with previous DTMRI data which showed a sharp transition in fiber direction between the endocardial and epicardial fiber layers (Pashakhanloo et al. 2016). The atrial anatomies were discretized using tetrahedral elements with a mean edge length of $\approx 238\,\upmu \hbox {m}$. This ensures at least two FEs across the myocardium of the LA, that can be as thin as $500\,\upmu \hbox {m}$ (Whitaker et al. 2016) and shows transmural variations in myofiber directions. The resulting FE meshes had 2.7, 1.8 and 1.1 million vertices and 14.8, 9.7 and 5.3 million elements, respectively.

### Biomechanics model

The myocardium of the LA was modeled as a nonlinear hyperelastic, nearly incompressible and transversely isotropic material. Consistent with previous cardiac mechanics models (Nash and Hunter 2000), we define two rectangular Cartesian coordinates $\mathbf {x}$ and $\mathbf {X}$. $\mathbf {x}$ defines the current location of a material point in the deformed configuration. $\mathbf {X}$ defines the location of a material point in the undeformed reference configuration. The deformation gradient $\mathbf{F}$, with $J = \mathrm{det}{} \mathbf{F} > 0$, describes the deformation of a continuum body from the reference configuration $\varOmega _0(\mathbf{X})$ to the current configuration $\varOmega _t(\mathbf{x})$. Furthermore, the right Cauchy–Green tensor $\mathbf{C} = \mathbf{F}^\mathrm{T}{} \mathbf{F}$ represents a deformation measure, while the Green strain tensor $\mathbf{E} = \frac{1}{2} (\mathbf{C} - \mathbf 1)$ represents a strain measure. The nearly incompressible myocardium of the LA was modeled using a multiplicative decomposition of the deformation gradient $\mathbf{F}$ (see, e.g., Flory 1961) according to $\mathbf{F} = J^{1/3}\overline{\mathbf{F}}$ and $\mathbf{C} = J^{2/3}\overline{\mathbf{C}}$, with $\mathrm{det}\overline{\mathbf{F}} = \mathrm{det}\overline{\mathbf{C}} = 1$. The mechanical deformation in the myocardium of the LA was governed by the quasi-static equilibrium equation given as

1 $$\begin{aligned} -\,\nabla \cdot \mathbf{F}{} \mathbf{S}(\mathbf{U},\mathbf{X},t) = \mathbf{0}, \end{aligned}$$

for $t\in \left[ 0,T\right]$, $T>0$, where $\mathbf{U}(\mathbf{X},t)$ is the displacement and $\mathbf{S}(\mathbf{U},\mathbf{X})$ is the second Piola-Kirchhoff stress tensor. In computational electromechanics (EM) simulations, the mathematical representations of cardiac electrophysiology (EP) and biomechanics (BM) are interconnected. The deformation of the myocardium in the LA is caused by imposed loads, e.g., intra-atrial pressure, and displacements, e.g., mitral valve ring motion, as well as the active mechanical contraction generated in the heart muscle. Thus, the total second Piola-Kirchhoff stress tensor $\mathbf{S}$ is additively decomposed according to

2 $$\begin{aligned} \mathbf{S} = \mathbf{S}_{\mathrm{p}} + \mathbf{S}_{\mathrm{a}}, \end{aligned}$$

where $\mathbf{S}_{\mathrm{p}}$ and $\mathbf{S}_{\mathrm{a}}$ are the passive and the active stress tensor, respectively. The corresponding Cauchy stress tensors $\varvec{\sigma }$, $\varvec{\sigma }_{\mathrm {p}}$ and $\varvec{\sigma }_{\mathrm {a}}$ are computed by the push-forward operation according to $\varvec{\sigma }_{(\bullet )}=J^{-1}\mathbf{F}{} \mathbf{S}_{(\bullet )}{} \mathbf{F}^\top$.

The passive stress tensor $\mathbf{S}_{\mathrm{p}}$ was calculated using the constitutive relation

3 $$\begin{aligned} \mathbf{S}_{\mathrm{p}} = 2\frac{\partial {\Psi }(\mathbf{C})}{\partial \mathbf{C}} \end{aligned}$$

where ${\Psi }$ is a transversely isotropic and invariant-based strain energy function. The strain energy function was composed of 3 individual functions

4 $$\begin{aligned} {\Psi }(\mathbf{C}) = {\Psi }_{{\mathrm{vol}}}(J) + {\overline{{\Psi }}}_{{\mathrm{iso}}}(\overline{{\mathbf{C}}}) + {{\Psi }_{{\mathrm {aniso}}}({\mathbf {C}}, {\mathbf {f}}_{0})}, \end{aligned}$$

where ${\Psi }_{\mathrm{vol}}$ is the volumetric contribution to the strain energy function and ${\overline{\Psi }}_{\mathrm{iso}}$ and ${\Psi }_{\mathrm{aniso}}$ are the isochoric contributions to the strain energy function referring to the isotropic and anisotropic parts, respectively. The prevailing myocyte orientation is denoted as $\mathbf {f}_0$. The volumetric contribution was modeled as

5 $$\begin{aligned} {\Psi }_{\mathrm{vol}}(J) = \frac{\mu }{2}\ln (J)^{2}, \end{aligned}$$

where $\mu > 0$ is a penalty parameter to enforce the incompressibility of the myocardium in the LA. The volumetric part of the strain energy function was modeled using the isotropic contribution (Demiray 1972)

6 $$\begin{aligned} \overline{{\Psi }}_{\mathrm{iso}}(\overline{\mathbf{C}}) = \frac{a}{2b}\{\exp [b(\bar{I}_{1}-3)]-1\}, \end{aligned}$$

with $\bar{I}_{1} = \mathrm{tr}\overline{\mathbf{C}}$, and the anisotropic contribution including the myofiber direction (Gasser et al. 2006)

7 $$\begin{aligned} {\Psi }_{\mathrm{aniso}}(\mathbf{C},\mathbf{f}_{0}) = \frac{a_{\mathrm {f}}}{2b_{\mathrm {f}}} \{\exp [b_{\mathrm {f}}(\kappa {I}_{1} + (1-3\kappa ){I}_{4}-1)^{2}] - 1\}, \end{aligned}$$

with ${I}_{4} = \mathbf{f}_{0}\cdot \mathbf{C}{} \mathbf{f}_{0}$. The constitutive model parameters in (6) and (7), i.e., *a*, *b*, $a_{\mathrm {f}}$ and $b_{\mathrm {f}}$, were considered positive and the structural parameter $\kappa \in [0,1/3]$, a phenomenological parameter in this case. Note, that we use an unsplit deformation gradient for the anisotropic contribution to minimize locking effects (Gültekin et al. 2019; Helfenstein et al. 2010; Sansour 2008). We test the dependence of the results on the penalty parameter $\mu$ in the sensitivity analysis (see Sect. 3.3). The proposed strain energy function represents a special case of the strain energy function in Eriksson et al. (2013) used to model the myocardium of the LV, modified to incorporate the transverse isotropic myocardial structure of the LA rather than the orthotropic myocardial structure of the LV. While the time step size for mechanics was $t_{\mathrm {mech}} = 1\,\text {ms}$, it was significantly smaller for EP, where $t_{\mathrm {EP}} = 25\,{\upmu \hbox {s}}$.

### Electrophysiology model

The cellular EP was described using the standard Courtemanche model (Courtemanche et al. 1998) to simulate the human atrial action potential in all patients. The modifications suggested in Cherry and Evans (2008) and Cherry et al. (2008), i.e., constant intracellular sodium and potassium, were implemented to prevent a transient model behavior. The intracellular current flow responsible for the spread of electrical activation in the atrial myocardium was calculated using the monodomain equation

8 $$\begin{aligned} \beta _{\mathrm{m}} C_{\mathrm{m}} \frac{\mathrm{d} V_{\mathrm{m}}}{\mathrm{d}t} + \beta _{\mathrm{m}} I_{\mathrm{ion}}(V_{\mathrm{m}},{\varvec{\eta }}) = \nabla \cdot ({\varvec{D}}_{\text {m}} \nabla V_{\text {m}}) + I_{\text {tr}}, \end{aligned}$$

where $\beta _{\text {m}}$ is the membrane surface-to-volume ratio, $C_{\text {m}}$ is the membrane capacity, $V_{\text {m}}$ is the transmembrane potential, $I_{\text {ion}}$ is the density of the total ionic currents as a function of $V_{\text {m}}$ and a set of state variables ${\varvec{\eta }}$, $I_{\text {tr}}$ is a transmembrane stimulus current and ${\varvec{D}}_{\text {m}}$ represents the monodomain conductivity tensor.

### Electromechanics model

The active stress tensor $\mathbf{S}_{\mathrm{a}}$ resulting from the mechanical contraction of the cardiac myocytes in the LA was assumed to act in the myofiber direction $\mathbf{f}_{0}$ and fiber dispersion is considered as in Eriksson et al. (2013). Thus,

9 $$\begin{aligned} &\hat{\mathbf{H}}_{\mathrm {a}}= \frac{\kappa }{1-2\kappa }{} \mathbf{C}^{-1} + \frac{1-3\kappa }{1-2\kappa }(\mathbf{f}_{0} \cdot \mathbf{C}\mathbf{f}_{0})^{-1}{} \mathbf{f}_{0}\otimes \mathbf{f}_{0},\nonumber \\& \mathbf{S_{\mathrm {a}}}(\mathbf{C}, \mathbf{f}_{0})= \hat{S}_{\mathrm {a}}\hat{\mathbf{H}}_{\mathrm {a}}, \end{aligned}$$

where $\hat{S}_{\mathrm {a}}$ is the scalar valued active stress generated in the cardiac myocytes and $\kappa \in [0,1/3]$ is the same dispersion parameter as in (7). The active stress $\hat{S}_{\mathrm{a}}$ was determined using a simplified form of the Niederer phenomenological active contraction model (Niederer et al. 2011b), without length dependence, given as

10 $$\begin{aligned} \hat{S}_{\mathrm{a}} = {\left\{ \begin{array}{ll} T_{\mathrm{peak}}\tanh ^{2}\left( \frac{t_{\mathrm{s}}}{\tau _{\mathrm{c}}}\right) \tanh ^{2}\left( \frac{t_{\mathrm{t}}-t_{\mathrm{s}}}{\tau _{\mathrm{r}}}\right) \mathrm{} &{} \text {if} \quad 0< t_{\mathrm{s}} < t_{\mathrm{t}}\\ 0 &{} \text {otherwise} \end{array}\right. } \end{aligned}$$

where $T_{\mathrm{peak}}$ is the peak isometric tension, $t_{\mathrm{s}}$ is the time after the onset of contraction, $t_{\mathrm{t}}$ is the duration of the active contraction and $\tau _{\mathrm{c}}$ and $\tau _{\mathrm{r}}$ are the contraction and relaxation time constants, respectively. The time after the onset of the contraction was calculated as

11 $$\begin{aligned} t_{\mathrm{s}} = t - t_{\mathrm{a}} - t_{\mathrm{d}}, \end{aligned}$$

with *t* denoting the finite element simulation time, $t_{\mathrm{a}}$ is the local activation time, defined when the local transmembrane potential passes the threshold voltage $V_{\mathrm {m,thresh}}$ and $t_{\mathrm{d}}$ is the electromechanical delay.

### Computational model parameters

#### Passive tissue biomechanics

Bellini et al. (2013) measured passive stiffness in tissue samples taken from anterior and posterior regions of the human LA. For each region, the material samples were studied under biaxial loading. In thin square samples, orthogonal distributed tensions were applied along each edge, where the square lay in the plane defined by directions 1 and 2 and direction 3 was out of plane. The ratio between tensions in the 1 and 2 directions were set to 1:0.5, 0.5:1, 1:0.75 and 0.75:1, as well as equiaxial loading, 1:1. The reference tension was set to 30 $\hbox {Nm}^{-1}$, such that a ratio of 1:0.5 corresponded to tensions of 30 $\hbox {Nm}^{-1}$ and 15 $\hbox {Nm}^{-1}$ in directions 1 and 2, respectively. We used this data to fit the parameters of our strain energy function given in Eq. 4.

In the posterior samples, we assumed that endocardial and epicardial fiber directions aligned with direction 1, corresponding to Green strain $E_{11}$ and stress $S_{11}$. In the anterior samples, we assumed that the fibers were orthogonal with the endocardial fibers running in direction 2, corresponding to Green strain $E_{22}$ and stress $S_{22}$ and with the epicardial fibers running in direction 1. The relative contribution of the endocardial and epicardial layers was defined as a free variable $\phi$, where a value of 1 would mean the stress was borne entirely by the endocardial layer. The strain energy function of the 2-layer material was given as (DeBotton 2005; DeBotton and Shmuel 2009)

12 $$\begin{aligned} {\Psi }(\mathbf{C}) = \phi {\Psi }_{\mathrm {endo}}(\mathbf{C}) + (1-\phi ) {\Psi }_{\mathrm {epi}}(\mathbf{C}), \end{aligned}$$

where ${\Psi }_{\mathrm{endo}}$ and ${\Psi }_{\mathrm{epi}}$ are the strain energy function for the endocardial and epicardial layers, respectively, each defined by Eq. 4. We assumed that both layers experience the same applied deformation. The second Piola-Kirchhoff stress tensor $\mathbf{S}$ for an incompressible material was calculated as

13 $$\begin{aligned} \mathbf{S} = - p_{\mathrm{h}} \mathbf{C}^{-1} + 2\frac{\partial {\Psi }(\mathbf{C})}{\partial \mathbf{C}}, \end{aligned}$$

where $p_{\mathrm{h}}$ is the Lagrange multiplier enforcing incompressibility. The expansion of the relation led to

14 $$\begin{aligned} \mathbf{S} = -\, p_{\mathrm{h}} \mathbf{C}^{-1} + 2\left( \phi \frac{\partial {\Psi }_{\mathrm {endo}}(\mathbf{C})}{\partial \mathbf{C}} + (1-\phi )\frac{\partial {\Psi }_{\mathrm {epi}}(\mathbf{C})}{\partial \mathbf{C}}\right) . \end{aligned}$$

The nonlinear least squares problem associated with the characterization of the mechanical material behavior in the LA was solved in MATLAB (The MathWorks, Inc., Natick, United States of America) subsequent to initial data cleaning. The objective function used in the minimization problem was given as

15 $$\begin{aligned} \arg \min _{{{{{\Phi }}}}} \, {\Gamma _{\mathrm{N}}}({{{{\Phi }}}}) = \sum _{i = 1}^{n} \left( {S}_{\mathrm{data}} - {S}_{\mathrm{model}}\right) ^{2}, \end{aligned}$$

where $S_{\mathrm {data}}$ is the measured stress, $S_{\mathrm {model}}$ is the simulated stress and *n* is the total number of data points recorded after data averaging including the different locations, i.e., anterior and posterior, directions, i.e., $S_{11}$ and $S_{22}$, and protocols and ${{{\Phi }}}$ is the material parameter set for the strain energy function.

**Fig. 1:**
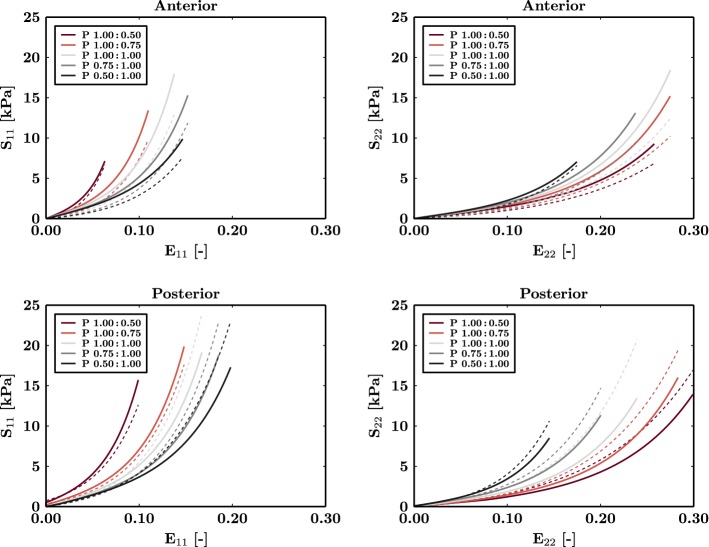
Constitutive law fitting. Comparison of model (solid) and experimental (dashed) passive material properties Experimental data from Bellini et al. (2013) for different tension ratios (P) in the 1 and 2 directions. $S_{11}$ is the stress in direction 1, $S_{22}$ is the stress in direction 2, $E_{11}$ is the Green strain in direction 1 and $E_{22}$ is the Green strain in direction 2

The dispersion parameter $\kappa$ was constrained according to $\kappa \in [0,1/3]$, corresponding to fibers all aligned in one direction and an isotropic model. The relative size of the endocardium parameter $\phi$ was constrained to fall between $\in [0.1,0.9]$ to ensure that both endocardial and epicardial fibers, that are known to be present in the atrium (Pashakhanloo et al. 2016), are included in the model. All other material parameters were constrained to be positive. The fitted strain energy function parameters are $a = 2.92\,\hbox {kPa}$, $b=5.6$, $a_{\mathrm {f}}=11.84\,{\hbox {kPa}}$, $b_{\mathrm {f}}=17.95$ and $\kappa =0.17$. As the optimal fitted value of $\phi$ is 0.1, this suggests that a smaller value of $\phi$ is possible. The fitted constitutive law is shown in Fig. 1. The sensitivity of the model results to the fitted values of *a*, $a_{\mathrm {f}}$ and $\kappa$ are included in our sensitivity analysis.

#### Passive biomechanics

The LA anatomy is recorded during diastasis when the ventricle and atria are close to relaxed. However, there is still an atrial pressure that could be as high as 1 kPa (Stefanadis et al. 1998). As the atria are very thin, relative to the ventricles, they will be more compliant and even this low pressure will cause the atria to deform. The thin walls make estimating the reference configuration using unloading techniques applied in the ventricles challenging as the atria are prone to buckling. For this reason, we used the measured anatomy as the reference configuration. This approximation results in the model operating at lower fiber strains where the material properties are more compliant (Nikou et al. 2016). To account for this decreased stretch, we scaled the stiffness by a factor of 2. We have tested the dependence of the results to this parameter in the sensitivity analysis (see Sect. 3.3).

#### Active biomechanics

Since model parameters scale with organ phenotypes, the parameters for the active contraction model were manually scaled to achieve the desired ejection fraction, peak pressure and contraction duration. The parameters were initialized to ventricular parameters (Niederer et al. 2011b). The final parameter set was $t_{\mathrm {d}}=10.0\,\text {ms}$, $T_{\mathrm {peak}}=50.0\,\text {kPa}$, $\tau _{c}=40.0\,\text {ms}$, $\tau _{\mathrm {r}}=110.0\,\text {ms}$ and $t_{\mathrm {t}}=300.0\,\text {ms}$. The membrane potential threshold for defining $t_{\mathrm {a}}$ was set to $V_{\mathrm {m,Thresh}}=-\,60.0\,\hbox {mV}$. More advanced techniques to personalize the parameterization, such as gradient, Latin hyper cube or grid search-based simulations, were computationally intractable due to the high computational cost of simulating atrial mechanics (approximately 1000 to 10,000 CPU core hours per simulation).

#### Electrophysiology

Single cell stimulation on the standard Courtemanche model (Courtemanche et al. 1998) using 1000 beats with a basic cycle length of 1000 ms was performed prior to EM simulations to obtain steady state conditions.

The conduction velocities in the LA were chosen as 1.20 and 0.40 m/s in longitudinal and transverse direction, respectively, leading to an anisotropy ratio of 3/1, well within the range of reported values for healthy patients (Dimitri et al. 2012; Kneller et al. 2002). To match the reported conduction velocities in the EP simulations, the monodomain conductivity tensor ${{\varvec{D}}}_{\mathrm{m}}$ was iteratively fitted using the method described in Mendonca Costa et al. (2013). The conductivity in the longitudinal ($D_{{\ell }}$) and transverse ($D_{\mathrm {t}}$) directions were set to 0.74 $\hbox {Sm}^{-1}$ and 0.08 $\hbox {Sm}^{-1}$, respectively. The membrane surface-to-volume ratio and the membrane capacity were set to standard values of $\beta _{\mathrm {m}}=1400\,\hbox {cm}^{-1}$ and $C_{\mathrm {m}}=1.0\,\upmu \hbox {Fcm}^{-2}$, respectively, for all simulations (Niederer et al. 2011a).

### Boundary conditions

#### Electrophysiology

The atrial electrophysiology model was activated by a stimulation applied on the epicardium in the vicinity of Bachmann’s Bundle for 2 ms with an amplitude of 500 $\upmu \hbox {Acm}^{-2}$ to approximate the physiological activation from the right atrium (Markides et al. 2003).

#### Biomechanics

The atrial cardiac cycle can be separated into three phases: first as a reservoir, where the atria stores blood during ventricular contraction. Second, as a conduit where the atria passively lets blood flow from the pulmonary veins to the ventricle. Thirdly as a pump, where the atria contracts to force blood into the ventricles.

Our simulations focus on the active contraction phase, when pressure and stress will be highest. The anatomy is derived from the cardiac CT images recorded during ventricular diastasis when the left atrium is in the conduit phase. We have taken this anatomy as an approximation of the reference anatomy.

The simulation was constrained by applying spring-like boundary conditions (Land and Niederer 2018) at the PVs and the MV, respectively. We initialize the model by inflating the atria from a zero pressure up to a pressure of 10 mmHg that reflects the mid pressure reached during atrial systole (Stefanadis et al. 1998; Ágoston et al. 2015). We have not implemented dynamic pressure volume boundary conditions for the pulmonary veins and the left ventricle.

The atrial mechanical boundary conditions are less sophisticated than the level expected in ventricular simulations and the current challenges and required developments are discussed below in the limitations section.

### Numerical framework

Left atrial electromechanics simulations were performed in CARP (Vigmond et al. 2003, 2008). CARP mechanics were previously verified against the cardiac mechanics N-version benchmark Land et al. (2015). The nonlinear mechanical problem was solved using Newton’s method until the minimum of the relative and the absolute norm of the residual vector reduced to $\varepsilon < 1\mathrm {e}-6$. For all linear subproblems, we used the generalized minimal residual (GMRES) method with algebraic multigrid (AMG) preconditioning and an error tolerance of $\varepsilon < 1\mathrm {e}-8$.

For more details on the preconditioned Krylov subspace methods see comprehensive research articles by Neic et al. (2012) and Augustin et al. (2016).

### Post-processing

#### Wall thickness calculation

Wall thickness in thin complex structures can be challenging to define Jones et al. (2000). For this reason, we previously applied the Laplace based wall thickness calculation method to the atria Bishop et al. (2016). This method was able to calculate wall thickness in the atria but is computationally expensive, especially when using very large meshes. Here, we use a faster method based in the eikonal equation. The eikonal equation was solved over the finite element meshes using a fast iterative method (Fu et al. 2013; Neic et al. 2017) where the wavefront was initiated on all nodes of the epicardium simultaneously given a constant isotropic conduction velocity. Local wall thickness was then computed from wavefront arrival times at the endocardium. This fast and robust approach was verified against results from Bishop et al. (2016).

#### Wall curvature calculation

To calculate wall curvature, we used a 3D sphere fitting approach similar to Thomas and Chan (1989). The algorithm is based on the minimization of the distance between the points of a endocardial surface patch and the radius of a fitted sphere in a least square sense. The local curvature is then defined as the inverse sphere radius. The endocardial surface patch was defined as a circular patch of elements of approximately 5 mm radius. Due to the fine, anatomically detailed FE meshes and the already very high complexity of the framework, curvature computations were based on the relatively simple but most widely applied Ji et al. (2015) approach using spheres. Note that more advanced geometric fittings using ellipses, hyperbolas, and parabolas, e.g., Ahn et al. (2001) and line integrals, e.g., Lin et al. (2010) are also proposed in the literature. However, these more sophisticated algorithms will also introduce additional complexity and parameters. We demonstrated in Sect. 2.9 that the correlations are robust to changes of the endocardial surface patch size. This suggests that the conclusions are not highly dependent on the curvature computation algorithm.

### Sensitivity analysis

To test the dependence of model findings on model parameters, the Spearman-$\rho$ coefficients between the wall thickness and curvature with wall stress were computed for different sets of parameters and compared to a control case (patient case 3). Simulations were run with the following parameters changed, where $\pm \,25$ % corresponds to an increase and a decrease of the respective parameter by 25 %:

1. Inflation pressure *p*$$\pm \,25$$ %;
2. Peak isometric tension $T_{\mathrm {peak}}$ in Eq. (10) $\pm \,25$ %;
3. Isotropic material parameter *a* in Eq. (6) $\pm \,25$ %;
4. Anisotropic material parameter $a_{\mathrm {f}}$ in Eq. (7) $\pm \,25$ %;
5. Stiffness Scale factor (stiff.). Scaling *a* in Eq. (6) and $a_{\mathrm {f}}$ in Eq. (7) each by $\pm \,25$ %;
6. Dispersion material parameter $\kappa$ in Eq. (7) $\pm \,25$ %;
7. Isotropic model with $\kappa =1/3$ in Eqs. (7) and (9);
8. Fully incompressible model with $1/\mu = 0$ in Eq. (5) and a block-system formulation;
9. Reduced penalty parameter $\mu =1000\,\text {kPa}$ and $\mu ={500}\,\text {kPa}$.

To calculate the sensitivity of the correlations with respect to the variables that were used to generate and analyze the model geometry, we calculate Spearman-$\rho$ coefficients for simulations with:

1. Curvature patch size (Sect. 2.8.2) $\pm \,25$ %;
2. Noised model: Gaussian noise (mean $\mu =0\,{\upmu \hbox {m}}$, standard deviation $\varsigma =100\,{\upmu \hbox {m}}$) was added to the initial geometry of patient case 3 and subsequently smoothed using ParaView (Ayachit 2015);
3. Constant thickness model: a mesh with constant thickness of 0.5 mm was generated based on the endocardial surface of patient case 3, using the software Gmsh (Geuzaine and Remacle 2009). Note, that the thickness related Spearman-$\rho$ values for this model are deliberately omitted.
4. 0 % cutoff: the whole region including boundary domains is used for correlation computations
5. 50 % cutoff: only the inner 50 % of the domain, measured as distance from the pulmonary inlets and the mitral valve ring, is used for correlation computations
6. 5/25 % cutoff: 5 % of the domain close to the pulmonary outlets and 25 % of the domain close to the mitral valve is not considered for the computations
7. 25/5 % cutoff: 25 % of the domain close to the pulmonary outlets and 5 % of the domain close to the mitral valve is not considered for the computations
8. $\varOmega _t$: the deformed domain is used for curvature and wall thickness computations;
9. stim. $\Gamma _{\mathrm {endo}}$: the whole endocardial surface is used for stimulation. Since cells are contracting simultaneously peak isometric tension $T_{\mathrm {peak}}$ in Eq. (10) had to be reduced by 25 %.

To calculate the sensitivity of the correlations with respect to the definition of stress, we calculate Spearman-$\rho$ coefficients where stress is defined as:

1. Principal stresses: let $\lambda _1$, $\lambda _2$ and $\lambda _3$ be the eigenvalues of the Cauchy stress tensor $\varvec{\sigma }$. Then
  $$\begin{aligned} \sigma ^{\mathrm {1st}}&=\max \{\lambda _1,\lambda _2,\lambda _3\}, \\ \sigma ^{\mathrm {3rd}}&=\min \{\lambda _1,\lambda _2,\lambda _3\}, \text{ and }\\ \sigma ^{\mathrm {2nd}}&={\text {tr}}(\varvec{\sigma }) - \sigma ^{\mathrm {1st}} - \sigma ^{\mathrm {3rd}} \end{aligned}$$
  are the principal stresses.
2. Fiber stress $$\sigma ^{\mathrm {f}}=\mathbf{f}_0 \cdot \varvec{\sigma }{} \mathbf{f}_0$$
3. Stress magnitude $$\left| \varvec{\sigma }\right| = \left( \varvec{\sigma }:\varvec{\sigma }\right) ^{1/2}$$
4. von Mises stress $\sigma ^{\mathrm {M}} = \left( 3/2\;\sigma '_{ij}\sigma '_{ij}\right) ^{1/2}$ and $\sigma '_{ij} = \sigma _{ij} - {1/3} \, \delta _{ij} \sigma _{kk}$ is the deviatoric stress.

Each of the above-mentioned stress measurements is computed for the total ($\varvec{\sigma }_{\mathrm {p}}+\varvec{\sigma }_{\mathrm {a}}$), passive ($\varvec{\sigma }_{\mathrm {p}}$) and active ($\varvec{\sigma }_{\mathrm {a}}$) Cauchy stress as well as for the total second Piola-Kirchhoff stress ($\varvec{S}_{\mathrm {p}}+\varvec{S}_{\mathrm {a}}$).

### Comparison to laplace estimates

To compare simulated stress calculations with the Law of Laplace, we consider an extension of the Laplace law (Mirsky and Parmley 1973) that takes into account the finite thickness of the wall and is based on the volume of the cavity after inflation ($V_{\mathrm {infl}}$) and the volume of the wall ($V_{\mathrm {wall}}$)

16 $$\begin{aligned} \sigma ^{\mathrm {La}}_{\mathrm {infl}} = \frac{p_{\mathrm {infl}}}{\left( \frac{V_{\mathrm {infl}} + V_{\mathrm {wall}}}{V_{\mathrm {infl}}}\right) ^{2/3}-1}, \end{aligned}$$

for the inflation pressure $p_{\mathrm {infl}}=10\,\mathrm{mmHg}$. For perfect spheres Eq. (16) is equivalent to the standard Laplace law for finite wall thickness, for more details see Gsell et al. (2018). Using Boyle–Mariotte’s law, we get for the cavity pressure at the fully contracted state $p_{\mathrm {cont}}$ using the measured volume of the cavity after contraction ($V_{\mathrm {cont}}$),

17 $$\begin{aligned} p_{\mathrm {cont}} = p_{\mathrm {infl}}V_{\mathrm {infl}} V_{\mathrm {cont}}^{-1}, \end{aligned}$$

and consequently

18 $$\begin{aligned} \sigma ^{\mathrm {La}}_{\mathrm {cont}} = \frac{p_{\mathrm {cont}}}{\left( \frac{V_{\mathrm {cont}} + V_{\mathrm {wall}}}{V_{\mathrm {cont}}}\right) ^{2/3}-1}. \end{aligned}$$

**Fig. 2:**
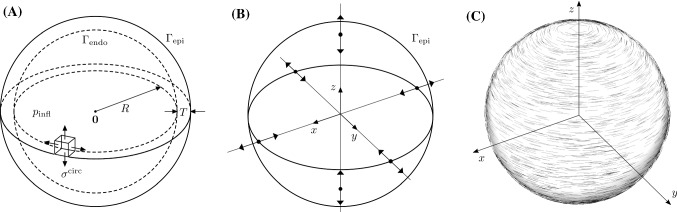
Comparison to Laplace estimates. **a** Geometric setup with *R* the inner radius and *T* the thickness of the spheres. A pressure $p_{\mathrm {infl}}$ was applied to the inner surface $\Gamma _{\mathrm {endo}}$. **b** Dirichlet boundary conditions were enforced at the intersections of the Cartesian axes with the outer surface $\Gamma _{\mathrm {epi}}$. Displacements at these points were restricted to be along the respective intersecting axes. **c** Transversely isotropic setup with one fiber family in the circumferential direction

The inner radius of the spheres was varied from $R\in \left\{ 20,25,30\right\}$ mm; the wall thickness was varied from $T\in \left\{ 0.5, 2.5, 5.0\right\}$ mm. For the geometric setup and the mechanical boundary conditions, see Fig. 2. Passive inflation and subsequent active contraction experiments were performed with the same simulation settings and parameters as described for the anatomical atria models. Three different fiber laws were applied to each of the spheres: *isotropic* with $\kappa =1/3$ in Eqs. (7) and (9); *transversely isotropic* (tr. iso.) with one fiber family in the circumferential direction, see Fig. 2c; *orthotropic* (ortho.) with two fiber families; the first, $\mathbf{f}_0^1$, in circumferential direction shown in Fig. 2c; the second, $\mathbf{f}_0^2$, in circumferential direction normal to $\mathbf{f}_0^1$. The anisotropic response (7) is used for each fiber direction, i.e.,

$$\begin{aligned} {\Psi }_{\mathrm{aniso}}(\mathbf{C}, \mathbf{f}_0^1, \mathbf{f}_0^2) = \frac{1}{2}\left( {\Psi }_{\mathrm{aniso}}(\mathbf{C},\mathbf{f}_0^1)+{\Psi }_{\mathrm{aniso}}(\mathbf{C},\mathbf{f}_0^2)\right) , \end{aligned}$$

and the active stress (9) for each fiber direction, i.e.,

$$\begin{aligned} \mathbf{S_{\mathrm {a}}}(\mathbf{C}, \mathbf{f}_0^1, \mathbf{f}_0^2) = \frac{1}{2}\left( \mathbf{S_{\mathrm {a}}}(\mathbf{C}, \mathbf{f}_0^1) + \mathbf{S_{\mathrm {a}}}(\mathbf{C}, \mathbf{f}_0^2)\right) . \end{aligned}$$

This type of fiber setting preserves the spherical shape of the geometry in the contracted state. For the thinnest spheres, ($T=0.5\,\text {mm}$) a *fully incompressible* (incomp.) case with $1/\mu = 0$ in Eq. (5) using locking-free finite elements and the orthotropic fiber law is also included.

## Results

### Reference anatomies and simulations

**Fig. 3:**
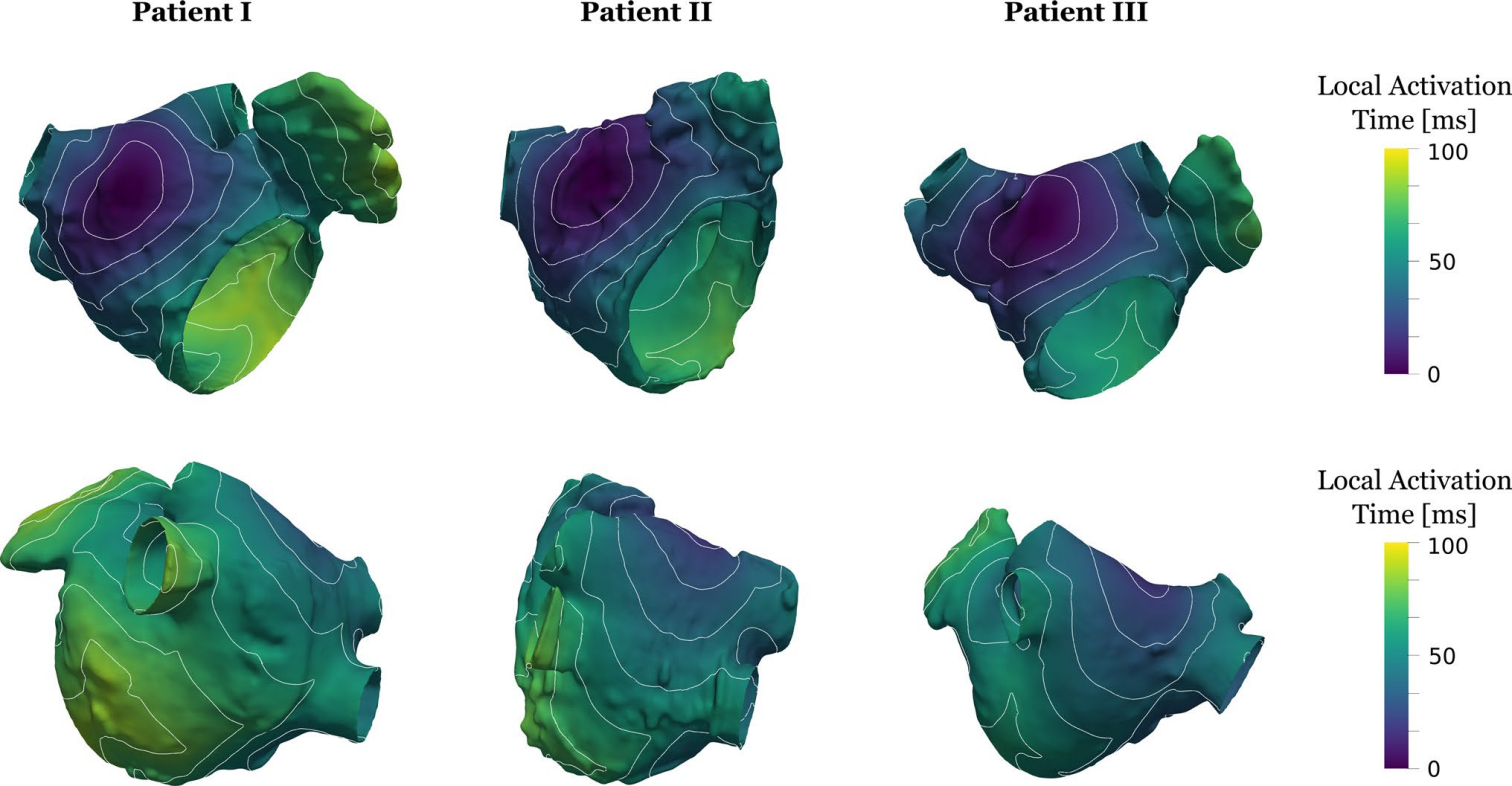
Model anatomies and activation patterns. Anterior (top row) and posterior (bottom row) perspective of patients showing the local activation time for the universal electrophysiology reference simulations. Isochrones are provided in 10 ms intervals

**Fig. 4:**
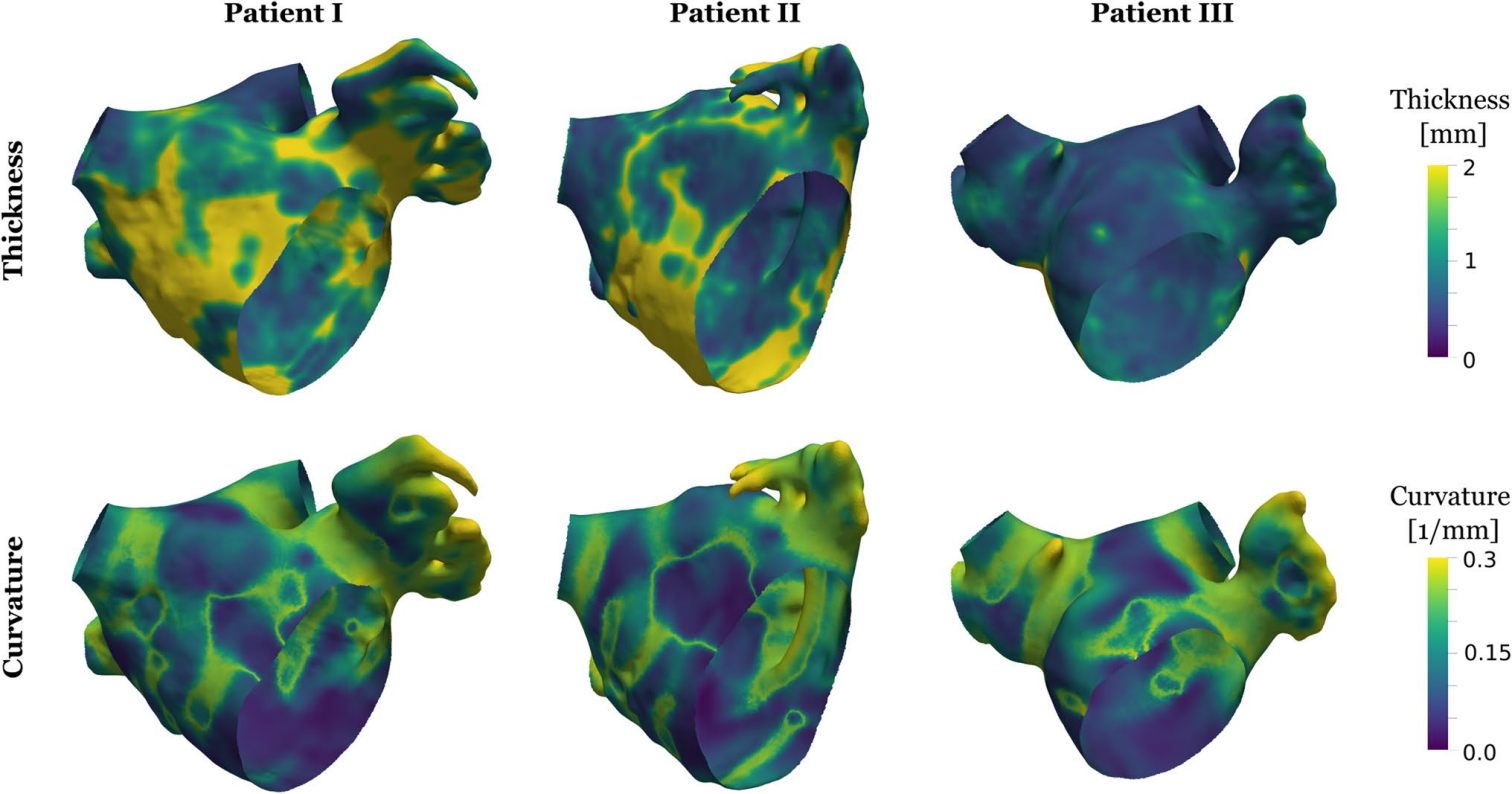
Curvature and thickness. Anterior perspective of analyzed patients showing the left atrial wall thickness in the first and curvature in the second row. Brighter colors correspond to larger thickness and curvature values

A summary of the patient characteristics is shown in Table 1. The reference model anatomies and the corresponding activation patterns are shown in Fig. 3 following the simulated sinus beat. The activation starts near Bachmann’s bundle on the anterior wall and spreads over the roof and around the mitral valve, with activation finishing in the appendage and posterior wall. The resulting distributions of curvature and thickness are shown in Fig. 4. The thickness pattern was distinct between patients, with patient III having a uniformly thinner atrium, compared to patients I and II. The curvature, as expected, was higher around the ostium of the pulmonary veins and appendage and lower curvature in general on the body of the LA. All models were inflated to a pressure of $p_{\mathrm {infl}}=$10 mmHg to approximate the mid atrial contraction pressure and contraction and activation were initiated to simulate atrial contraction under a fixed atrial pressure.

**Table 1:** Summary attributes of patient attributes

Index	Sex	Age	Comorbidities
1	M	35	HLD
2	F	48	
3	F	54	PAF, SSS

*M* male, *F* female, *HLD* hyperlipidemia, *PAF* paroxysmal atrial fibrillation, *SSS* sick sinus syndrome

**Fig. 5:**
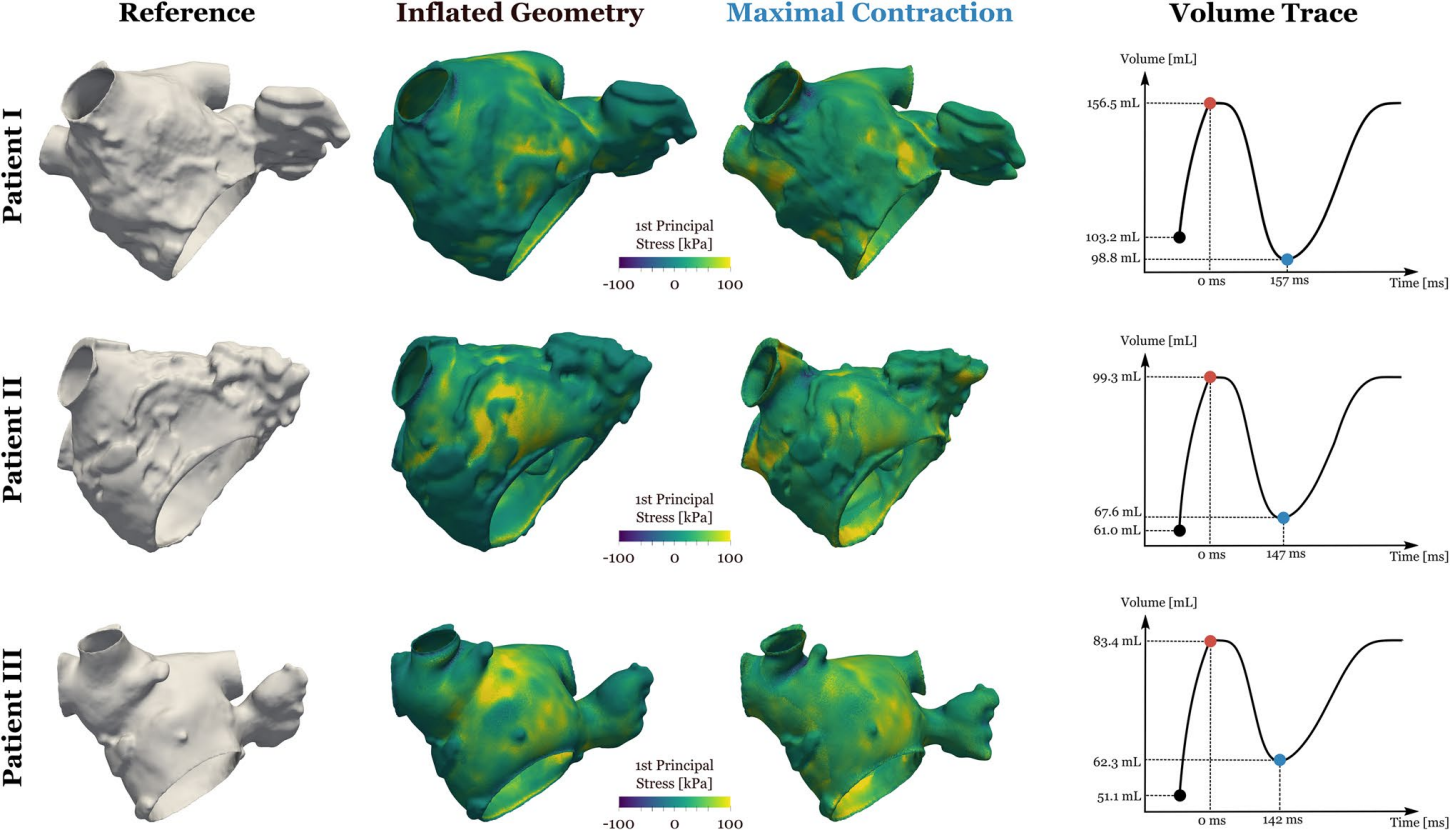
Stress fields. Anterior perspective of analyzed patients showing the reference geometry and the first principal total Cauchy stress ($\varvec{\sigma }_{\mathrm {p}}+\varvec{\sigma }_{\mathrm {a}}$) for the biomechanics simulations at the inflated and maximally contracted state. To the right, the volume curve over time for the inflation to 10 mmHg, and subsequent active contraction and relaxation, for each patient

The atrial anatomy and corresponding stress fields are shown in Fig. 5. Table 2 summarizes the changes in volume during the simulation.

The wall thickness and endocardial wall curvature were calculated on the reference mesh. Median wall thickness is $1.07\,\pm \,0.47$ mm, $0.86\, \pm \, 0.39$ mm and 0.64 ± 0.15 mm and median curvature is $0.167\,\pm \,0.082\,{\hbox {mm}^{-1}}$, $0.172\,\pm \, 0.077\,{\hbox {mm}^{-1}}$ and $0.169\,\pm \, 0.078\,\hbox {mm}^{-1}$ in patient cases, 1, 2 and 3, respectively, where ± values indicate the interquartile range.

**Table 2:** Summary LA volume changes during simulated atrial contraction

*i*	$V_{0}$ (ml)	$V_{\mathrm {infl}}$ (ml)	$V_{\mathrm {cont}}$ (ml)	IF (%)	EF (%)
1	101.03	156.47	98.81	154.9	36.9
2	61.04	99.31	67.57	162.2	32.0
3	51.11	83.36	62.32	163.1	25.2

*i* Case index; $V_0$ reference volume; $V_{\mathrm {infl}}$ volume after inflation, prior to atrial contraction; $V_{\mathrm {cont}}$ minimum atrial volume at contracted state; inflation fraction (IF) is $V_{\mathrm {infl}}/V_0$; and ejection fraction (EF) is ($V_{\mathrm {infl}} - V_{\mathrm {cont}})/V_{\mathrm {infl}}$

### Correlation of wall stress with local anatomy

**Fig. 6:**
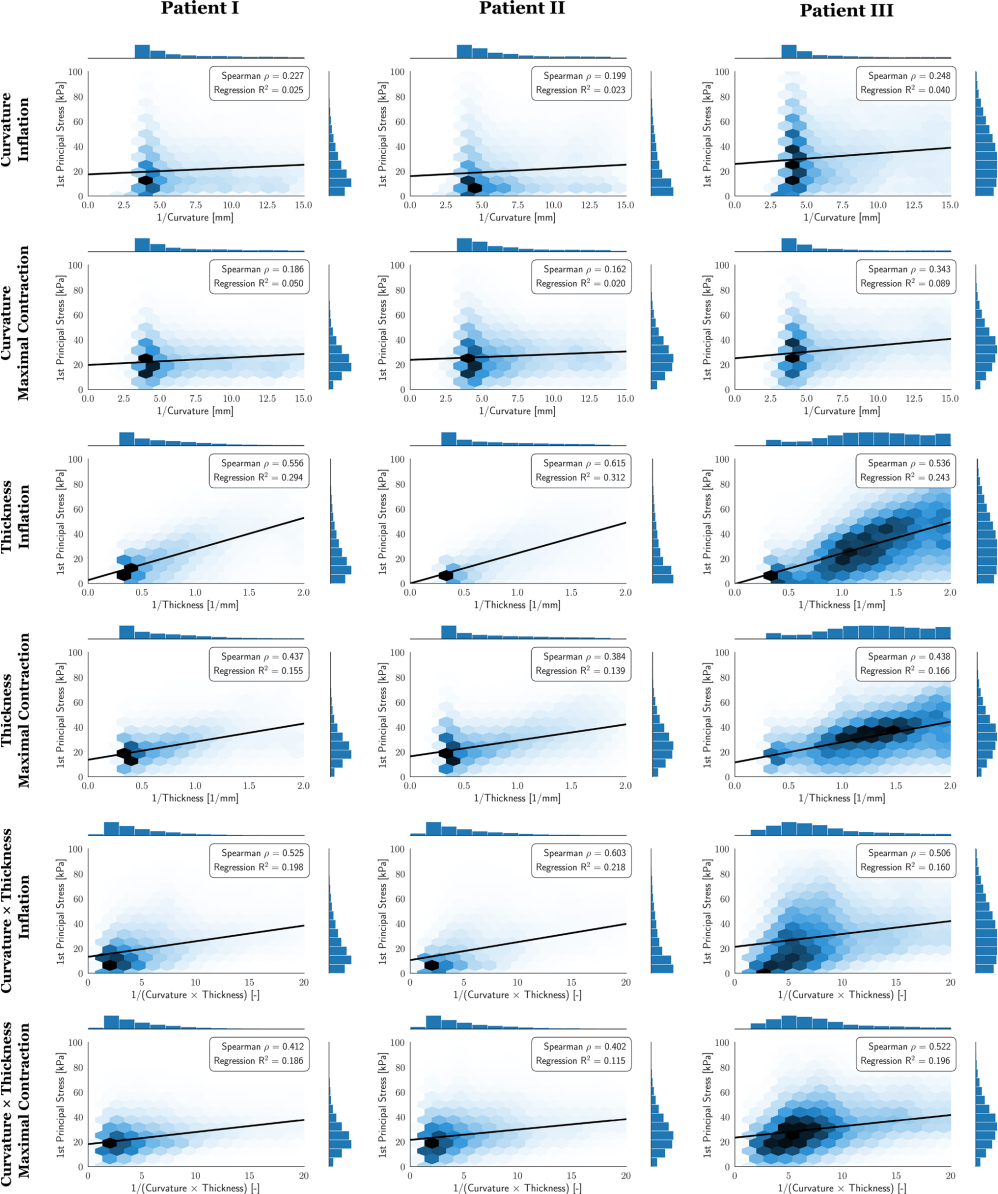
Hexbin plots of (i) total stress ($\varvec{\sigma }_{\mathrm {p}}+\varvec{\sigma }_{\mathrm {a}}$) vs. the inverse of curvature, (ii) total stress versus the inverse of thickness and (iii) total stress versus the inverse of (curvature $\times$ thickness) for the inflated and the maximal contracted state, respectively. Thickness, curvature and the first principal stress were interpolated on the nodes of the finite element mesh and analyzed. Regions close to the pulmonary vein inlets and the mitral valve were excluded from the statistical analysis and the plots since data there may be affected by the spring-type boundary conditions. Black lines show a linear regression model fit. Spearman’s $\rho$ values and $R^2$ values from the linear regression fit are given for each plot

In the Law of Laplace, wall stress is proportional to the radius of curvature (or the inverse of curvature) and inversely proportional to wall thickness. We calculate the principal wall stress following passive inflation and at the point of maximal contraction from Fig. 5 for each case. We have plotted these two stress fields against the reciprocal of curvature and the reciprocal of wall thickness. The normality of the curvature, stress and wall thickness distributions was tested using Kolmogorov–Smirnov tests. As all distributions were not normally distributed, which is also indicated by the histograms in Fig. 6, we made comparisons using a Spearman’s correlation coefficient. This represents a minimum test to demonstrate a monotonic relationship, consistent with the stress being dependent on the corresponding input parameter.

Correlations are calculated for each plot in Fig. 6. When comparing curvature and wall stress we find a weak but consistent correlation during inflation (0.20–0.25) and maximal contraction (0.16–0.34). A stronger correlation is found between wall thickness and stress during inflation (0.54–0.62), however, this also decreases with maximal contraction (0.38–0.44). As wall stress is proportional to the ratio of the radius of curvature and wall thickness in the Law of Laplace it seemed possible that the wall stress may have a higher correlation with the inverse product of wall thickness and curvature. However, the correlation of the inverse of wall thickness and curvature was only slightly different from the correlation between wall stress and wall thickness during inflation (0.51–0.60) and contraction (0.40–0.42).

The correlation statistics between the principal wall stress against wall thickness or curvatures are summarized in Table 3. To test if the correlations between wall thickness and curvature with wall stress were affected by a relationship between wall thickness and curvature, we calculated the correlation of wall thickness and curvature and found no meaningful correlation (− 0.027 to 0.12) in the reference domain.

**Table 3:** Summary, Spearman’s correlations between the principle wall Cauchy stress and the curvature and wall thickness on the reference grid

*i*	$\rho ^{\textsf {Wt}}_{\mathrm {infl}}$	$\rho ^{\textsf {Wt}}_{\mathrm {cont}}$	$\rho ^{\textsf {C}}_{\mathrm {infl}}$	$\rho ^{\textsf {C}}_{\mathrm {cont}}$	$\rho ^{\textsf {WtC}}_{\mathrm {infl}}$	$\rho ^{\textsf {WtC}}_{\mathrm {cont}}$	$\rho ^{\textsf {Wt}}_{\mathrm {C}}$
1	0.556	0.437	0.227	0.186	0.525	0.412	0.005
2	0.615	0.384	0.199	0.162	0.603	0.402	− 0.027
3	0.536	0.438	0.248	0.343	0.506	0.424	0.117

$\rho ^{\textsf {Wt}}_{ {\bullet }}$ are Spearman’s correlations between stress and inverse of wall thickness, $\rho ^{\textsf {C}}_{{\bullet }}$ are Spearman’s correlations between stress and inverses of curvature, $\rho ^{\textsf {WtC}}_{ {\bullet }}$ are Spearman’s correlations between stress and inverse of (curvature $\times$ thickness). The subscripts $\mathrm {infl}$ and $\mathrm {cont}$ denote the inflated and maximal contracted state, respectively. $\rho ^{\textsf {Wt}}_{\mathrm {C}}$ are Spearman’s correlations between wall thickness and curvature. *p*-values were $<\,0.001$ for all cases

### Dependence of wall stress and local anatomy correlations on model parameters

All three patient cases had qualitatively similar correlations across all values tested. None of the correlations were higher than 0.62. To allow for a larger number of simulations, we preformed an extensive analysis on Case 3 to determine what model parameters play a role in the relationship between local anatomy, encoded in wall thickness and curvature, and wall stress, see Table 4.

**Table 4:** Sensitivity to parameter modifications

Parameter	$V_{\mathrm {infl}}$ (mL)	$V_{\mathrm {cont}}$ (mL)	$t_{\mathrm {cont}}$ (s)	IF (%)	EF (%)	$\rho ^{\textsf {Wt}}_{\mathrm {infl}}$	$\rho ^{\textsf {Wt}}_{\mathrm {cont}}$	$\rho ^{\textsf {C}}_{\mathrm {infl}}$	$\rho ^{\textsf {C}}_{\mathrm {cont}}$	$\rho ^{\textsf {WtC}}_{\mathrm {infl}}$	$\rho ^{\textsf {WtC}}_{\mathrm {cont}}$
Control	83.4	62.3	142	163.1	25.2	0.536	0.438	0.248	0.343	0.506	0.522
$p_{\mathrm {infl}}+25\%$	85.7	69.7	144	167.6	18.6	0.540	0.488	0.250	0.346	0.510	0.553
$p_{\mathrm {infl}}-25\%$	80.4	52.9	141	157.3	34.2	0.528	0.337	0.243	0.320	0.498	0.446
$T_{\mathrm {peak}}+25\%$	83.4	54.6	140	163.1	34.4	0.536	0.372	0.248	0.334	0.506	0.477
$T_{\mathrm {peak}}-25\%$	83.4	69.8	144	163.1	16.2	0.536	0.486	0.248	0.333	0.506	0.542
$a+25\%$	81.9	61.6	141	160.3	24.8	0.541	0.440	0.245	0.342	0.506	0.523
$a-25\%$	85.1	63.3	142	166.5	25.7	0.530	0.436	0.251	0.343	0.505	0.521
$a_{\mathrm {f}}+25\%$	82.6	62.3	142	161.6	24.6	0.535	0.436	0.252	0.342	0.508	0.520
$a_{\mathrm {f}}-25\%$	84.3	62.4	141	164.9	26.0	0.538	0.441	0.243	0.343	0.504	0.523
stiff. $+25\%$	81.2	61.6	142	158.9	24.2	0.539	0.438	0.248	0.342	0.508	0.521
stiff. $-25\%$	86.1	63.4	141	168.5	26.4	0.531	0.438	0.247	0.343	0.503	0.522
$\kappa +25\%$	85.7	67.0	143	167.7	21.8	0.547	0.474	0.232	0.339	0.501	0.540
$\kappa -25\%$	81.3	59.8	141	159.2	26.5	0.525	0.408	0.261	0.341	0.510	0.502
$\kappa =0$ for $\mathbf{S}_{\mathrm {a}}$	83.4	52.4	140	163.1	37.2	0.537	0.346	0.248	0.327	0.507	0.457
$\kappa =1/3$	90.4	89.1	144	176.8	1.4	0.560	0.560	0.203	0.201	0.487	0.486
$1/\mu =0$	83.5	62.5	142	163.3	25.1	0.553	0.457	0.251	0.348	0.519	0.538
$\mu =1000$	83.9	62.3	142	164.1	25.8	0.558	0.475	0.258	0.375	0.526	0.569
$\mu =500$	84.5	62.1	142	165.3	26.5	0.562	0.482	0.261	0.382	0.530	0.579

Shown are inflated volume $V_{\mathrm {infl}}$; contracted volume $V_{\mathrm {cont}}$; time of maximal contraction $t_{\mathrm {cont}}$; inflation fraction (IF), ejection fraction (EF). Spearman-$\rho$ values for correlation: inverse of wall thickness ($\textsf {Wt}$), inverse of curvature ($\textsf {C}$), inverse of (thickness $\times$ curvature) ($\textsf {WtC}$) each versus stress at inflation ($\rho ^{\bullet }_{\mathrm {infl}}$) and contraction ($\rho ^{\bullet }_{\mathrm {cont}}$). For all models the initial volume was $\approx$ 51 mL since they are all based on the patient case 3 from Table 1.

To test if the degree of deformation affected the anatomical wall stress correlations, we recalculated the correlations in simulations with altered endocardial pressure and altered active contraction, that will alter deformation. Changes in deformation due to pressure and active contraction caused small (< 0.01) changes in the correlation of wall thickness or curvature with wall stress during inflation. During contraction, changes in deformation due to changes in pressure and active contraction caused small ($<\,0.03$) changes in the correlation of curvature with wall stress, but caused larger ($>0.1$) changes in the correlation of wall thickness and wall stress. We then tested if the isotropic stiffness, anisotropic stiffness, stiffness scaling factor or degree of anisotropy affected the anatomical wall stress correlations. None of the correlations experienced large changes with the greatest change being from 0.44 to 0.47. We tested limit cases of fiber dispersion: active tension acting only in the fiber direction and isotropic fiber distribution. In the active contraction case, the isotropic fiber distribution acts as a hydro-static pressure, so there is limited deformation; this made wall thickness and wall curvature have the same correlation with stress in the inflation and contraction cases and results in a limited ejection fraction. Active stress only acting in the fiber direction had no affect, as expected, on the inflation correlation and decreased the correlations in the contraction case. Changes in incompressibility caused minor changes in the inflation correlations but increasing the degree of incompressibility caused a decrease in the contraction correlations.

### Dependence of wall stress and local anatomy correlations on model creation and analysis parameters

In addition to the model parameters, additional variables were set to create and analyze the model geometry. We tested if these factors play a role in determining the local anatomical - wall stress correlations, see Table 5. The curvature at each mesh vertex is calculated from a local region of elements defined by a patch. Increasing and decreasing the size of this patch caused minor (<0.05) change in the correlations. Introducing noise into the mesh to see if the specific anatomy was important caused minor (<0.05) changes in the correlations. Removing the effects of wall thickness by setting wall thickness to be constant caused an increase from 0.248 to 0.343 and 0.343 to 0.467 in the correlation of curvature with wall stress during inflation and contraction, respectively.

**Table 5:** Sensitivity to geometrical modifications

Parameter	$V_{\mathrm {infl}}$ (mL)	$V_{\mathrm {cont}}$ (mL)	$t_{\mathrm {cont}}$ (s)	IF (%)	EF (%)	$\rho ^{\textsf {Wt}}_{\mathrm {infl}}$	$\rho ^{\textsf {Wt}}_{\mathrm {cont}}$	$\rho ^{\textsf {C}}_{\mathrm {infl}}$	$\rho ^{\textsf {C}}_{\mathrm {cont}}$	$\rho ^{\textsf {WtC}}_{\mathrm {infl}}$	$\rho ^{\textsf {WtC}}_{\mathrm {cont}}$
Control	83.4	62.3	142	163.1	25.2	0.536	0.438	0.248	0.343	0.506	0.522
Patch $+25\%$	83.4	62.3	142	163.1	25.2	0.536	0.439	0.248	0.352	0.522	0.535
Patch $-25\%$	83.4	62.3	142	163.1	25.2	0.536	0.438	0.250	0.339	0.491	0.511
Noised	80.6	60.9	141	157.7	24.5	0.512	0.432	0.293	0.380	0.481	0.510
Constant	82.7	68.4	145	161.8	17.3	–	–	0.343	0.467	–	–
0% cutoff	83.4	62.3	142	163.1	25.2	0.495	0.410	0.202	0.330	0.464	0.505
50% cutoff	83.4	62.3	142	163.1	25.2	0.582	0.523	0.344	0.400	0.584	0.587
25/5% cutoff	83.4	62.3	142	163.1	25.2	0.510	0.453	0.312	0.345	0.541	0.530
5/25% cutoff	83.4	62.3	142	163.1	25.2	0.606	0.506	0.260	0.369	0.536	0.565
$\varOmega _t(\mathbf {x})$	83.4	62.315	142	163.1	25.2	0.561	0.518	0.390	0.446	0.525	0.600
stim. $\Gamma _{\mathrm {endo}}$	83.358	72.176	162	163.1	13.4	0.536	0.498	0.238	0.326	0.506	0.544

Shown are inflated volume $V_{\mathrm {infl}}$; contracted volume $V_{\mathrm {cont}}$; time of maximal contraction $t_{\mathrm {cont}}$; inflation fraction (IF), ejection fraction (EF). Spearman-$\rho$ values for correlation: inverse of wall thickness ($\textsf {Wt}$), inverse of curvature ($\textsf {C}$), inverse of (thickness $\times$ curvature) ($\textsf {WtC}$) each versus stress at inflation ($\rho ^{\bullet }_{\mathrm {infl}}$) and contraction ($\rho ^{\bullet }_{\mathrm {cont}}$)

In the analysis presented above, we have excluded regions where we applied boundary constraints; when these are included the correlations all decrease. To test if boundary conditions play a role in the correlations, we only considered the middle 50% of the anatomy that is remote from regions where boundary conditions are applied (pulmonary veins and mitral valve). The local wall thickness and curvature to wall stress correlations in these regions were all higher than correlations measured across the whole atria, suggesting boundary conditions decrease the correlations between local anatomy and wall stress. Excluding tissue preferentially near the mitral valve or the pulmonary veins shows that the mitral valve has the greater impact on the correlation. We find that using the deformed, as opposed to the reference, anatomy for calculating wall thickness and curvature improves all correlations. Finally, we demonstrated that the activation pattern did not play a large role in the correlations. Stimulating the entire endocardium causes the correlations to change by − 0.01 to 0.06.

### Dependence of wall stress and local anatomy correlations on stress definition

In this study we defined wall stress as the first principal component of the combined active and passive stress, as this provides a general coordinate free measure of local stress. To test the role of the stress definition used in the anatomical wall stress correlations we observed, we recalculated the correlations using the total, active or passive stress in the fiber direction, the stress magnitude and the von Mises stress, see Table 6. The correlations for the total stress show minimal differences for the different stress definitions. In the case of the active and passive stress there was limited change in the correlation between wall thickness and curvature with any of the stress definitions during inflation. The stress magnitude and von Mises stress had lower and higher correlations with the passive and active stress, respectively during contraction.

**Table 6:** Sensitivity to stress measurements

Stress type	Stress part	$\rho ^{\textsf {Wt}}_{\mathrm {infl}}$	$\rho ^{\textsf {Wt}}_{\mathrm {cont}}$	$\rho ^{\textsf {C}}_{\mathrm {infl}}$	$\rho ^{\textsf {C}}_{\mathrm {cont}}$	$\rho ^{\textsf {WtC}}_{\mathrm {infl}}$	$\rho ^{\textsf {WtC}}_{\mathrm {cont}}$
1st principal stress	$\varvec{\sigma }_{\mathrm {p}}+\varvec{\sigma }_{\mathrm {a}}$	0.536	0.438	0.248	0.343	0.506	0.522
2nd principal stress	$\varvec{\sigma }_{\mathrm {p}}+\varvec{\sigma }_{\mathrm {a}}$	0.415	0.290	0.323	0.320	0.495	0.419
3rd principal stress	$\varvec{\sigma }_{\mathrm {p}}+\varvec{\sigma }_{\mathrm {a}}$	0.041	0.089	0.192	0.178	0.170	0.186
fiber stress	$\varvec{\sigma }_{\mathrm {p}}+\varvec{\sigma }_{\mathrm {a}}$	0.522	0.410	0.252	0.366	0.492	0.517
Stress magnitude	$\varvec{\sigma }_{\mathrm {p}}+\varvec{\sigma }_{\mathrm {a}}$	0.578	0.480	0.266	0.370	0.547	0.574
Von Mises stress	$\varvec{\sigma }_{\mathrm {p}}+\varvec{\sigma }_{\mathrm {a}}$	0.552	0.448	0.207	0.290	0.483	0.484
1st principal stress	$\varvec{\sigma }_{\mathrm {p}}$	0.536	0.310	0.248	0.307	0.506	0.424
2nd principal stress	$\varvec{\sigma }_{\mathrm {p}}$	0.415	0.288	0.323	0.251	0.495	0.352
3rd principal stress	$\varvec{\sigma }_{\mathrm {p}}$	0.041	0.273	0.192	0.266	0.170	0.378
Fiber stress	$\varvec{\sigma }_{\mathrm {p}}$	0.522	0.402	0.252	0.299	0.492	0.471
Stress magnitude	$\varvec{\sigma }_{\mathrm {p}}$	0.578	− 0.244	0.266	− 0.206	0.547	− 0.322
Von Mises stress	$\varvec{\sigma }_{\mathrm {p}}$	0.552	0.126	0.207	0.135	0.483	0.171
1st principal stress	$\varvec{\sigma }_{\mathrm {a}}$	–	0.222	–	0.172	–	0.259
2nd principal stress	$\varvec{\sigma }_{\mathrm {a}}$	–	0.086	–	0.063	–	0.077
3rd principal stress	$\varvec{\sigma }_{\mathrm {a}}$	–	− 0.096	–	− 0.034	–	$-$0.067
Fiber stress	$\varvec{\sigma }_{\mathrm {a}}$	–	0.214	–	0.269	–	0.314
Stress magnitude	$\varvec{\sigma }_{\mathrm {a}}$	–	0.401	–	0.317	–	0.462
Von Mises stress	$\varvec{\sigma }_{\mathrm {a}}$	–	0.285	–	0.206	–	0.318
1st principal stress	$\varvec{S}_{\mathrm {p}}+\varvec{S}_{\mathrm {a}}$	0.553	0.374	0.249	0.333	0.517	0.483
2nd principal stress	$\varvec{S}_{\mathrm {p}}+\varvec{S}_{\mathrm {a}}$	0.388	0.315	0.308	0.280	0.469	0.399
3rd principal stress	$\varvec{S}_{\mathrm {p}}+\varvec{S}_{\mathrm {a}}$	0.041	0.145	0.149	0.266	0.133	0.289
Fiber stress	$\varvec{S}_{\mathrm {p}}+\varvec{S}_{\mathrm {a}}$	0.532	0.390	0.272	0.358	0.515	0.504
Stress magnitude	$\varvec{S}_{\mathrm {p}}+\varvec{S}_{\mathrm {a}}$	0.595	0.456	0.282	0.393	0.569	0.578
Von Mises stress	$\varvec{S}_{\mathrm {p}}+\varvec{S}_{\mathrm {a}}$	0.576	0.375	0.174	0.185	0.474	0.365

Shown is total Cauchy stress ($\varvec{\sigma }_{\mathrm {p}}+\varvec{\sigma }_{\mathrm {a}}$), passive Cauchy stress ($\varvec{\sigma }_{\mathrm {p}}$), active Cauchy stress ($\varvec{\sigma }_{\mathrm {a}}$), and total second Piola-Kirchhoff stress ($\varvec{S}_{\mathrm {p}}+\varvec{S}_{\mathrm {a}}$). For each the stress magnitude, first, second, and third principal stress, fiber stress, and von Mises stress is computed. Spearman-$\rho$ values for correlation: inverse of wall thickness ($\textsf {Wt}$), inverse of curvature ($\textsf {C}$), inverse of (thickness $\times$ curvature) ($\textsf {WtC}$) each versus stress at inflation ($\rho ^{\bullet }_{\mathrm {infl}}$) and contraction ($\rho ^{\bullet }_{\mathrm {cont}}$). Active stress is 0 at the point of inflation, hence, results are omitted

### Testing the impact of geometric complexity on the correlation of wall stress with local anatomy

We have shown that degree of deformation, heterogeneous wall thickness, boundary conditions and choice of reference frame can all affect anatomical wall stress correlations. To confirm that these findings are not an artifact of the simulation code, we verified that the simulation code can replicate the Law of Laplace in idealized models. Using the same models, we tested if the fiber material model plays a role in the relationship between curvature and wall thickness. We performed simulations in an idealized sphere model of the atria with isotropic transversely isotropic and anisotropic fibers in a compressible and incompressible model with different wall thicknesses and curvature. In the case of the isotropic material, contraction is inhibited as the increase in active tension in all directions is equivalent to an increase in hydro-static pressure. Hence, the very low ejection fractions ($<2\,\%$) for the isotropic cases are to be expected. With the exception of the transversely isotropic case, passive circular stress results of the FE simulations $\overline{\sigma }^{\mathrm {circ}}_{\mathrm {infl}}$ match well with the Laplace estimates $\sigma ^{\mathrm {La}}_{\mathrm {infl}}$, (see Bold value’s columns in Table 7), especially for the thinnest spheres. As in these cases the Laplace laws are known to be almost exact, this serves as a code verification of the FE implementation. For the transversely isotropic case, the sphere is less resistant to strain in *z*-axis direction, see Fig. 2c. This results in a ellipsoidal shape after inflation and at contracted state and as a consequence Laplace estimates are less accurate. While the Laplace law is relatively precise in predicting passive stresses at inflation, consistent with the complex anatomical models, the estimates compare poorly in the active contracted state, in particular for anisotropic materials.

**Table 7:** Laplace law

Fibers	*R* (mm)	*T* (mm)	$V_{\mathrm {wall}}$ ($\hbox {cm}^{3}$)	$V_0$ (ml)	$V_{\mathrm {infl}}$ (ml)	$V_{\mathrm {cont}}$ (ml)	IF (%)	EF (%)	$\overline{\sigma }^{\mathrm {1st}}_{\mathrm {infl}}$ (kPa)	$\overline{\sigma }^{\mathrm {1st}}_{\mathrm {cont}}$ (kPa)	$\overline{\sigma }^{\mathrm {circ}}_{\mathrm {infl}}$ (kPa)	$\overline{\sigma }^{\mathrm {circ}}_{\mathrm {cont}}$ (kPa)	$\sigma ^{\mathrm {La}}_{\mathrm {infl}}$ (kPa)	$\sigma ^{\mathrm {La}}_{\mathrm {cont}}$ (kPa)
isotropic	20	0.5	2.6	33.5	59.7	58.6	178.0	1.8	53.3	52.8	** 46. 4**	45.9	** 46. 6**	46.6
tr. iso.	20	0.5	2.6	33.5	57.0	43.7	170.2	23.4	52.8	42.8	** 48. 4**	40.8	** 44. 6**	44.7
ortho.	20	0.5	2.6	33.5	56.0	49.9	167.3	11.0	50.5	44.8	** 44. 0**	39.0	** 43. 8**	43.9
incomp.	20	0.5	2.6	33.5	55.9	50.5	166.9	9.6	50.4	47.0	** 43. 9**	41.0	** 43. 7**	43.8
isotropic	20	2.5	14.2	33.5	48.6	47.7	145.0	1.7	8.7	7.9	** 7. 5**	6.7	** 7. 1**	7.2
tr. iso.	20	2.5	14.2	33.5	46.3	22.9	138.2	50.5	8.5	12.8	** 7. 7**	8.6	** 6. 8**	7.1
ortho.	20	2.5	14.2	33.5	45.5	22.0	136.0	51.7	8.1	5.1	**7. 0**	3.2	** 6. 7**	7.0
isotropic	20	5.0	31.9	33.5	44.5	43.8	133.0	1.6	4.0	3.1	** 3. 4**	2.5	** 3. 1**	3.1
tr. iso.	20	5.0	31.9	33.5	42.5	21.7	126.9	48.9	3.9	13.0	** 3. 5**	4.2	** 2. 9**	3.2
ortho.	20	5.0	31.9	33.5	41.8	20.7	124.8	50.5	3.7	4.3	** 3. 1**	− 1.1	** 2. 9**	3.1
isotropic	25	0.5	4.0	65.4	119.9	117.7	183.2	1.8	68.5	68.0	** 59. 7**	59.2	** 60. 2**	60.2
tr. iso.	25	0.5	4.0	65.4	114.5	98.4	175.0	14.1	67.9	60.7	** 62. 3**	57.5	** 57. 5**	57.5
ortho.	25	0.5	4.0	65.4	112.4	104.2	171.7	7.3	64.9	60.1	** 56. 5**	52.3	** 56. 4**	56.5
incomp.	25	0.5	4.0	65.4	112.0	104.8	171.2	6.4	64.8	61.9	** 56. 4**	54.0	** 56. 3**	56.3
isotropic	25	2.5	21.7	65.4	97.5	95.8	149.0	1.7	11.1	10.3	** 9. 7**	8.8	** 9. 3**	9.3
tr. iso.	25	2.5	21.7	65.4	93.0	45.7	142.2	50.8	10.9	13.3	** 10. 0**	10.3	** 8. 9**	9.2
ortho.	25	2.5	21.7	65.4	91.5	43.9	139.9	52.1	10.5	5.8	** 9. 0**	4.6	** 8. 8**	9.1
isotropic	25	5.0	47.6	65.4	89.5	87.0	136.8	1.7	5.1	4.2	** 4. 4**	3.5	** 4. 1**	4.1
tr. iso.	25	5.0	47.6	65.4	85.3	43.0	130.4	49.5	5.0	12.7	** 4. 5**	5.5	** 3. 9**	4.1
ortho.	25	5.0	47.6	65.4	83.8	41.2	128.2	50.8	4.7	4.5	** 4. 0**	0.3	** 3. 8**	4.1
isotropic	30	0.5	5.8	113.1	212.1	208.3	187.5	1.8	84.0	83.7	** 73. 2**	72.9	** 74. 1**	74.1
tr. iso.	30	0.5	5.8	113.1	202.3	183.3	178.9	9.4	83.4	78.0	** 76. 6**	73.7	** 70. 7**	70.7
ortho.	30	0.5	5.8	113.1	198.3	187.5	175.3	5.4	79.5	75.2	** 69. 3**	65.5	** 69. 3**	69.3
incomp.	30	0.5	5.8	113.1	197.5	188.2	174.7	4.7	79.5	76.9	** 69. 2**	67.0	** 69. 0**	69.1
isotropic	30	2.5	30.7	113.0	172.2	169.2	152.4	1.8	13.7	12.9	** 11. 9**	11.0	** 11. 5**	11.5
tr. iso.	30	2.5	30.7	113.0	164.4	80.9	145.5	50.8	13.4	14.1	** 12. 2**	11.9	** 11. 0**	11.3
ortho.	30	2.5	30.7	113.0	161.9	77.3	143.2	52.3	12.9	6.9	** 11. 1**	5.9	** 10. 9**	11.2
isotropic	30	5.0	66.5	113.0	158.2	155.5	139.9	1.7	6.3	5.4	** 5. 4**	4.5	** 5. 1**	5.1
tr. iso.	30	5.0	66.5	113.0	150.7	75.4	133.3	50.0	6.1	12.6	** 5. 5**	6.6	** 4. 8**	5.1
ortho.	30	5.0	66.5	113.0	148.2	72.3	131.1	51.2	5.8	4.7	** 5. 0**	1.4	** 4. 8**	5.0

Comparison of FE-based mean principal stresses ($\overline{\sigma }^{\mathrm {1st}}_{\mathrm {infl}}$, $\overline{\sigma }^{\mathrm {1st}}_{\mathrm {cont}}$) and mean circumferential fiber stresses ($\overline{\sigma }^{\mathrm {circ}}_{\mathrm {infl}}$, $\overline{\sigma }^{\mathrm {circ}}_{\mathrm {cont}}$) with Laplace estimations ($\sigma ^{\mathrm {La}}_{\mathrm {infl}}$, $\sigma ^{\mathrm {La}}_{\mathrm {cont}}$) for the inflated (infl.) and the fully contracted (cont.) state. Radius (*R*), which is the inverse of curvature *C*, and thickness (*T*) of the spheres are varied. Three different fiber laws are applied to each of the resulting spheres: *isotropic*, *transversely isotropic* (tr. iso.), and *orthotropic* (ortho.). For the thinnest spheres, ($T={0.5}\,\text {mm}$) a *fully incompressible* (incomp.) case is included. Additionally, wall volume $V_{\mathrm {wall}}$, initial cavity volume $V_0$, inflated cavity volume $V_{\mathrm {infl}}$ and cavity volume at contracted state $V_{\mathrm {cont}}$ are given as well as inflation fraction (IF) and ejection fraction (EF)

In bold are the circumferential fiber stresses and Laplace estimations for the inflated state; in these cases the Laplace laws are known to be almost exact

### Testing if the Law of Laplace can be used to estimate mean wall stress in the left atria

**Fig. 7:**
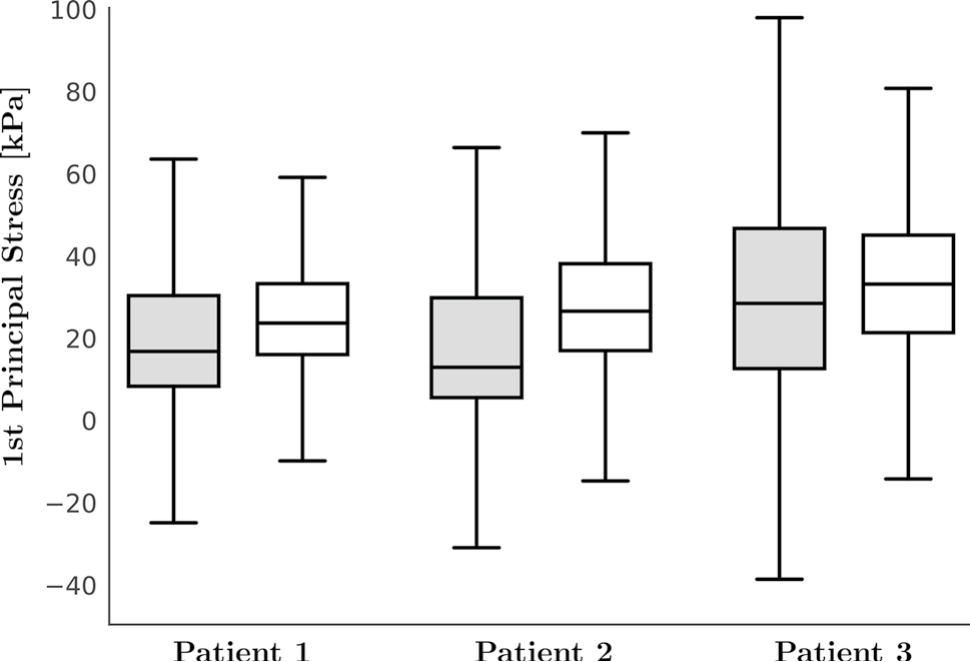
Statistical distribution of stress for all three patient cases. Boxplots show the distribution of the 1st principal Cauchy stress: in gray stresses at inflated ($\varvec{\sigma }^{\mathrm {1st}}_{\mathrm {infl}}$), in white stresses at contracted state ($\varvec{\sigma }^{\mathrm {1st}}_{\mathrm {cont}}$). The box represents the interquartile range (IQR) between lower quartile (25 %) and upper quartile (75 %); horizontal black line represents the median value; whisker ends represent the lowest and highest data points still within 1.5 IQR of the lower and upper quartiles, respectively

To test if the Law of Laplace can be used for estimating wall stress in the LA, we first calculated the stress distribution in the LA for all three cases, using the reference simulations (Fig. 7) and compared these distributions to the mean wall stress estimated using the Law of Laplace (Table 8). This shows that the Law of Laplace underestimates mean wall stress by 14–16% during passive inflation and 22–38% during active contraction. There is also a high degree of variation in the wall stress, under all conditions, that is not captured in the Law of Laplace wall stress estimate. Secondly, we calculated the maximum correlation achievable under ideal conditions in all three patient cases, where the factors identified in Case 3 that caused the greatest increase in correlations between wall stress and local anatomy were applied. Table 9, shows the correlations calculated where we quantified local anatomy in the deformed anatomy, with increased endocardial pressure, with decreased active tension and only considering the middle 50% of the atria. These factors are cumulative and cause an increase in correlations in all three cases, suggesting that these findings are not specific to Case 3. In particular, we note higher correlation with the inverse product of wall thickness and curvature: 0.61–0.68 for the inflated and 0.64–0.70 for the fully contracted case. The conditions imposed to maximize correlations, all increase the cavity volume and reduce the effect of boundary conditions; thus, higher correlations are to be expected as this setting is suited better to the Law of Laplace.

**Table 8:** Laplace law

Value	Unit	Patient 1		Patient 2		Patient 3	
		infl.	cont.	infl.	cont.	infl.	cont.
Min	(kPa)	− 573.01	− 336.46	− 515.86	− 635.81	− 416.53	− 381.09
Max	(kPa)	2304.60	422.54	869.39	749.39	389.49	357.67
Mean	(kPa)	21.01	25.51	19.92	28.16	30.93	33.69
SD	(kPa)	20.51	17.02	24.47	19.92	25.21	20.01
$\sigma ^{\mathrm {La}}$	(kPa)	17.67	17.85	17.19	17.34	26.30	26.40

Patient cases. Comparison of FE-based mean principal stresses (minimal, maximal, mean, and standard deviation) with Laplace estimations ($\sigma ^{\mathrm {La}}$), see Eqs. (16)–(18), for the inflated (infl.) and the fully contracted (cont.) state

**Table 9:** Summary, Spearman’s correlations between the principle wall Cauchy stress and the curvature and wall thickness where we quantified local anatomy in the deformed anatomy, increased endocardial pressure, decreased active tension and only considered the middle 50% of the atria

*i*	$\rho ^{\textsf {Wt}}_{\mathrm {infl}}$	$\rho ^{\textsf {Wt}}_{\mathrm {cont}}$	$\rho ^{\textsf {C}}_{\mathrm {infl}}$	$\rho ^{\textsf {C}}_{\mathrm {cont}}$	$\rho ^{\textsf {WtC}}_{\mathrm {infl}}$	$\rho ^{\textsf {WtC}}_{\mathrm {cont}}$
1	0.550	0.636	0.402	0.407	0.642	0.669
2	0.600	0.616	0.411	0.406	0.682	0.639
3	0.598	0.644	0.367	0.480	0.608	0.699

Abbreviations: $\rho ^{\textsf {Wt}}_{{\bullet }}$ are Spearman’s correlations between stress and inverse of wall thickness, $\rho ^{\textsf {C}}_{ {\bullet }}$ are Spearman’s correlations between stress and inverses of curvature, $\rho ^{\textsf {WtC}}_{{\bullet }}$ are Spearman’s correlations between stress and inverse of (curvature $\times$ thickness). The subscripts $\mathrm {infl}$ and $\mathrm {cont}$ denote the inflated and maximal contracted state, respectively. *p*-values were $<0.001$ for all cases

## Discussion

We have developed a modeling framework for simulating left atrium contraction. We have simulated passive inflation and active contraction of the atria. We have shown that, consistent with the Law of Laplace, the principal wall stress is dependent on LA wall thickness and curvature under conditions of passive inflation, with a higher dependence on wall thickness. Under conditions of active contraction we find a smaller correlation between wall stress and curvature or wall thickness. This finding is replicated in both idealized and complex geometries. A sensitivity analysis demonstrated that the correlations are robust to many model simulation parameters, model creation and analysis parameters and the definition of stress (Tables 4, 5, 6). To maximize the correlation of wall stress and local anatomy required calculations of wall thickness and curvature on the deformed geometry, only consider tissue that is remote from boundary conditions and when deformations are reduced.

In the model, we predicted maximal LA volumes of 83–156 mL following passive inflation. The final pressure was higher than expected under physiological conditions leading to higher volumes but close to reported values of $80\,\pm \,30$ mL in controls and $115\,\pm \,33$ mL in AF patients (Stojanovska et al. 2014). The ejection fractions predicted by the model were 25–37%, which are consistent with measurements of $\approx 30\%$ (Rodevand et al. 1999; Stefanadis et al. 1998). This shows that the model is capable of operating within a physiological range consistent with clinical observations.

A correlation was found between wall stress and both wall thickness and curvature during passive inflation (Fig. 6). This shows that both anatomical attributes are important. Wall thickness does have a stronger correlation (0.54–0.62) than curvature (0.20 to 0.25) emphasizing the importance of accounting for atrial wall thickness in personalized calculations of local wall stress. This may be particularly important when studying how local wall stress is correlated with local tissue remodeling.

In the Law of Laplace, wall stress is proportional to the ratio of the radius of curvature (the inverse of curvature) and the wall thickness. To test if this ratio had a stronger correlation with wall stress than wall thickness or curvature independently, we plotted the inverse product of wall thickness and curvature. This resulted in negligible improvement in the correlation (Fig. 6).

In the ventricles, myocardial wall stress plays a role in regulating growth and oxygen demand (Yin 1981) and was first associated with cardiac shape by Woods (1892). However, these relationships have not been evaluated in the atrium, where measuring wall thickness across the entire atria has only recently become possible (Bishop et al. 2016). We found that wall thickness had a greater impact on determining wall stress than curvature in the three patients cases studied. These three cases span a range of atrial pathologies from hyperlipidemia, which is associated with elevated blood pressure, atrial fibrillation, which is associated with a decrease in atrial mechanical function and a healthy control.

Increased left ventricle pressure is hypothesized to cause cellular hypertrophy and increased wall thickness to bring wall stress back to normal levels (Grossman et al. 1975). This is the first study to show that in the atria wall thickness correlates with local principal wall stress and similar regulatory pathways, that are hypothesized in the ventricle, may be present in the atria. Previous studies have also found that atrial wall stress is correlated with remodeling, in the form of fibrosis (Hunter et al. 2012), although this study was performed with homogeneous wall thickness and may need to be confirmed in models that account for varying wall thickness across the atrial body.

The potential link between curvature, wall thickness, wall stress, growth and fibrosis may have important, albeit complex, interactions with atrial electrophysiology. This becomes particularly important if remodeling in the atria are regulated by wall stress, as proposed in the ventricle (Grossman et al. 1975), and provides a possible link between increased arrhythmia risk and pathological changes in atrial loading. Previous studies have found atrial wall thickness and curvature impact the conduction velocity (Rossi et al. 2018), gradients in thickness are associated with stabilizing re-entrant activation patterns (Yamazaki et al. 2012) and patients with thicker atria are at higher risk of developing arrhythmias (Whitaker et al. 2016). Studying the interaction between wall stress, anatomy and atrial arrhythmias using computational simulations will require large highly detailed complex models and motivates further investment in multi-physics simulators and simulation speed.

To identify the factors that are important in determining the correlation between wall stress with wall thickness or curvature we preformed three sensitivity studies investigating model parameters, variables for creating and analyzing the model geometry and the definition of stress. For the vast majority of model perturbations, there was no to limited changes in the correlations suggesting that these are robust to model assumptions. We identified the choice of reference frame, degree of incompressibility, amount of deformation and boundary conditions as confounding factors in the correlation of local anatomy with wall stress. However, when including all of these factors in the model the maximum correlation was only 0.6–0.7, showing that in complex atrial anatomy it is unlikely that a simple anatomical-based law can be used to estimate local wall stress.

### Limitations

This is the first study of atrial mechanics to account for varying wall thickness derived from clinical images. We have applied our modeling framework to three patient cases, demonstrating that the techniques are applicable beyond a single case study. However, in contrast to work in the ventricles (Nordsletten et al. 2011; Augustin et al. 2016) we have applied a simple active contraction model, static boundary conditions and we have not unloaded the geometry.

The model of active contraction is driven by a phenomenological model of tension development to estimate atrial cellular contraction. We have used a simplified contraction model that would benefit from increased physiological detail. Previous attempts at modeling atrial contraction in organ scale models have adapted human ventricular models (Land and Niederer 2018) or used models initially fitted to rat ventricular data (Moyer et al. 2015; Adeniran et al. 2015). To improve simulations of atrial contraction will require the development of a model of human atrial contraction from detailed human atrial experimental measurements.

The passive mechanics parameters used in the simulations were dependent on two modeling decisions. First, in fitting the passive material properties the value of $\phi$ was fixed to fall within 0.1 and 0.9, to ensure that the model included both an endocardium and epicardium layer. The final fitted value was 0.1, suggesting that the optimal $\phi$ value could fall between 0 and 0.1. This constraint may have affected the fitted passive stiffness parameters. Second, the atria was not unloaded at the time point when the reference anatomy was created. As the atria exhibit nonlinear constitutive properties, the loaded reference geometry will results in smaller calculated strains. To compensate for these effects, we scaled the passive mechanics model by a factor of 2. To test if these decisions impacted the calculated correlations we altered the isotropic, fiber or combined stiffness (Table 4). None of these changes caused large differences in the correlations suggesting these model assumptions do not affect the study conclusions.

In the performed simulations, a biophysical cell model was used that simulates the full action potential and calcium dynamics. These models are more complex than may be necessary for our simulations. However, the relatively small cost of using a full cell model allows us to better capture the effects, if any, of wave curvature and activation speed. As electrophysiology was not the focus of the study, we did not investigate what effect the use of a biophysical cell model had on simulation results.

We have simulated a single phase of the atrial cardiac cycle against a fixed pressure boundary condition. To capture wall thickness in our model, we derived the model anatomy from cardiac CT images. While CT gives excellent resolution, to capture motion with cardiac CT requires a higher radiation dose and was not available for our patient cases. This meant that we did not have information on volume transients nor did we have information on mitral valve ring motion. Further, we did not have access to invasive pressure measurements or echocardiogram Doppler flow measurements to allow us to estimate atrial pressure. As a result, we were unable to simulate the reservoir phase of the atrial cycle that is driven by ventricular motion and we were unable to personalize dynamic boundary conditions so used literature values to define the fixed pressure boundary conditions.

Large deformation mechanics models are initiated from a reference unloaded geometry. The heart is never in an unloaded state as it always has a cavity pressure. To account for the cavity pressure, when the heart is imaged in patient-specific ventricular models, the meshes are unloaded to estimate the reference configuration based on the boundary conditions and the imaged deformed anatomy. This results in the myocardium being under strain when the heart is re-inflated back to the imaged volume. Due to the thin shell of the atria, unloading the atria risks buckling that would lead to an unstable simulation. The unloading of the atria may require specific numerical techniques to allow for a stable simulation. The absence of unloading would make the atria more compliant as it would operate at lower strains. To account for this potential underestimation of atrial stiffness, we increased the stiffness in our simulations. Accounting for atrial unloading will be important to enable the mapping of detailed-ex-vivo measurements into simulations of the atria in-vivo.

This study used publicly available patient-specific anatomies for three cases. This small sample does not represent the full variation in atria anatomies and limits the ability to generalize these findings to other patients. However, the correlations that we identified were qualitatively similar across all three cases in the reference simulations and all three cases saw similar increases in correlations under idealized conditions (Table 9). In addition, we were able to replicate the decreased correlation between local anatomy with wall stress during contraction in a sphere model (Table 7) observed in the reference models (Table 3). Two of the patients had known pathologies and were likely taking corresponding medication that have the potential to alter atrial electrophysiology and/or contraction. As models were generated from anonymized data, the drug history of each patient was not available for this study.

## Conclusion

We have created the first cohort of atrial mechanics models personalized to patients anatomy, including wall thickness. We found that the principal wall stress was determined more by the wall thickness than the curvature, necessitating personalized wall thickness measurements for calculating local wall stress. For the conditions considered here, the Law of Laplace provides a poor estimate of local wall stress in the left atrium. The choice of reference frame, degree of incompressibility, amount of deformation and boundary conditions were the main confounding factors, but did not fully explain the difference between the simulated wall stress and the Law of Laplace. This simulation framework provides a platform for studying the link between local anatomy, mechanics and electrophysiology.
